# Crosstalk between Macrophages and Pancreatic β-Cells in Islet Development, Homeostasis and Disease

**DOI:** 10.3390/ijms22041765

**Published:** 2021-02-10

**Authors:** Cristina Cosentino, Romano Regazzi

**Affiliations:** 1 Department of Fundamental Neurosciences, University of Lausanne, Rue du Bugnon 9, CH-1005 Lausanne, Switzerland; cristina.cosentino@unil.ch; 2 Department of Biomedical Sciences, University of Lausanne, Rue du Bugnon 7, CH-1005 Lausanne, Switzerland

**Keywords:** pancreatic islet, macrophage, diabetes

## Abstract

Macrophages are highly heterogeneous and plastic immune cells with peculiar characteristics dependent on their origin and microenvironment. Following pathogen infection or damage, circulating monocytes can be recruited in different tissues where they differentiate into macrophages. Stimuli present in the surrounding milieu induce the polarisation of macrophages towards a pro-inflammatory or anti-inflammatory profile, mediating inflammatory or homeostatic responses, respectively. However, macrophages can also derive from embryonic hematopoietic precursors and reside in specific tissues, actively participating in the development and the homeostasis in physiological conditions. Pancreatic islet resident macrophages are present from the prenatal stages onwards and show specific surface markers and functions. They localise in close proximity to β-cells, being exquisite sensors of their secretory ability and viability. Over the years, the crucial role of macrophages in β-cell differentiation and homeostasis has been highlighted. In addition, macrophages are emerging as central players in the initiation of autoimmune insulitis in type 1 diabetes and in the low-grade chronic inflammation characteristic of obesity and type 2 diabetes pathogenesis. The present work reviews the current knowledge in the field, with a particular focus on the mechanisms of communication between β-cells and macrophages that have been described so far.

## 1. Main

The crosstalk between pancreatic β-cells and macrophages has been commonly investigated in relation to islet inflammation during diabetes pathogenesis. In autoimmune type 1 diabetes (T1D), macrophages seem to play a crucial role in attracting autoreactive T-cells, finally triggering islet inflammation called “insulitis” [1]. Insulitis leads to β-cell demise and finally to β-cell loss. Interestingly, low-grade chronic inflammation has been associated with obesity and type 2 diabetes (T2D). In this scenario, macrophages are the main immune cells involved [2]. However, besides the role exerted in distressful pathological conditions, macrophages have been shown to be essential for normal tissue development and homeostasis. Macrophages’ phenotypes and signalling mechanisms are highly tissue-specific and depend on cues received from surrounding cells and milieu.

Here we review the role of macrophages in pancreatic β-cell development, homeostasis, and dysfunction, focusing on the signalling mechanisms mediating the crosstalk between the two cell types.

## 2. Macrophage Heterogeneity, Plasticity and Signalling

Macrophages are highly heterogeneous cells, virtually present in all tissues, deriving from the differentiation of circulating monocytes, which in turn originate from bone marrow myeloid precursors, common to other leucocytes and dendritic cells [3]. Circulating monocytes can migrate from bone marrow to different tissues, where they differentiate in macrophages. Although the model of monocyte derivation of macrophages has been widely accepted for years, recent technologies allowing cell fate tracing and single cell profiling revealed that subpopulations of macrophages develop prenatally, in early embryogenesis, from hematopoietic progenitors of the yolk sac [4]. In the prenatal stages, these cells migrate to constitute pools of tissue-resident macrophages, such as Langerhans cells and microglia, residing in the epidermis and the central nervous system, respectively [5,6]. These findings were corroborated in mouse models in which a lineage of myeloid cells was shown to be independent from the expression of Myb, a transcription factor essential for the development of hematopoietic stem cells [7]. In Myb knock out embryos, CD45^+^ F4/80^low^ CD11b^high^ macrophages were completely absent in different organs, indicating their hematopoietic origin, while CD45^+^ F4/80^high^ CD11b^low^ macrophages developed normally in the skin, spleen, pancreas, kidney, and lung [7]. Myb-independent macrophages derive from yolk sac precursors. Interestingly, pancreatic F4/80^high^ macrophages were found to reside in pancreatic islets in close proximity to β-cells [7]. However, the yolk sac origin of macrophages in human tissues is still under debate due to the difficulties in confirming the observations collected in mouse models. Once established, the pool of tissue-resident macrophages is maintained by local proliferation as well as by circulating monocytes migrating and differentiating in the different tissues [8].

For a long time, macrophages have been mainly described as effectors of the immune response, because of their functions in phagocytosis and their aptitude to present antigens to T-cells. However, tissue-resident macrophages possess crucial tissue homeostatic functions, such as the removal of cellular debris and apoptotic cells, independently from the activation of immune responses [8]. Clearance processes are crucial for tissue homeostasis and do not trigger inflammatory reactions. In addition, macrophages exert tissue- and niche-specific functions, developing peculiar phenotypes as a result of cues received from the environment [4]. In the adipose tissue, for instance, macrophages are responsible for regulating lipid and energy metabolism [9] and modulating insulin sensitivity [10]. Another source of macrophage heterogeneity derives from their high plasticity, permitting them to respond to stimuli elicited by the environment by switching their metabolic activation state. In an over-simplistic model, macrophage phenotypes are classified as pro-inflammatory (M1) and anti-inflammatory (M2); according to this interpretation, tissue-resident macrophages belong to the M2 class. Despite the vast variety of phenotypes, M2 macrophages are generally dependent on pro-survival and self-renewal stimuli that they receive from surrounding cells and milieu, such as the macrophage colony-stimulating factor (M-CSF), glucocorticoids, and cytokines, such as interleukin (IL)-4 and IL-13 produced by T helper 2 (Th2) cells and regulatory T-cells [11]. Other signals are specific to the tissue and niche where M2 macrophages promote trophic and clearance functions. On the other hand, M1 or classically activated macrophages are generally induced in response to interferon-γ (IFN-γ), which is produced during innate immune responses from T helper 1 (Th1) cells and CD8^+^ T cells or in response to tumour-necrosis factor (TNF) released by antigen-presenting cells (APCs) [12]. During inflammation, the macrophage number increases because of the recruitment of circulating monocytes. However, recent evidence suggests a major contribution of local proliferation of resident macrophages [13,14,15]. By rapidly switching their activation state in response to environmental stimuli, macrophages contribute to the initiation, progression, and resolution of inflammation. Interestingly, these cells are able to maintain their high plasticity even after being activated; for this reason, multiple intermediate activation states can be found concurrently. Furthermore, since tissue-resident macrophages have particular phenotypes depending on their origin and differentiation niche, they may present specific metabolic modulation and activation in response to the same stimulus. This makes the generalisation of macrophage phenotypes an obsolete model and suggests that studies at tissue and niche level should be conducted in order to characterise the specific macrophage metabolic profile, function, and interaction with surrounding cells.

Soluble factors, such as cytokines, are the most characterised signalling molecules in the crosstalk between macrophages and their target cells. Cytokines and chemokines are low molecular weight proteins binding to specific cell surface receptors and activating intracellular signalling pathways. Their action depends on the expression of receptors and on the proximity of target cells. Indeed, cytokines generally exert autocrine or paracrine effects, while only few of them have endocrine function, acting on longer distances, such as TNFα and M-CSF [16]. According to their activation state, macrophages produce different sets of cytokines. Pro-inflammatory cytokines include TNFα, IL-1, IL-6, IL-8, and IL-12. The pro-inflammatory response is crucial for host defence against damage and pathogens promoting cytotoxic responses, but the release of these cytokines can trigger inflammatory and autoimmune disorders. IL-10 is released by anti-inflammatory-activated macrophages to counteract the inflammatory state. This cytokine inhibits the expression of IFNγ from Th1 cells and suppresses the production of pro-inflammatory cytokines [16]. Direct cell-to-cell contact and extracellular vesicles are also necessary for the immunomodulatory mechanisms exerted by macrophages. Macrophage-derived extracellular vesicles can deliver proteins, metabolites, and nucleic acids to close or distant cells and have been implicated in the pathogenesis of cardiovascular, respiratory, and metabolic diseases [17].

## 3. Involvement of Macrophages in β-Cell Differentiation, Proliferation and Homeostasis

Macrophages are present in the pancreas from the embryonic state onwards. Although little is known about their role in islet morphogenesis, several observations highlighted their importance in the development of endocrine pancreas and, in particular, that of β-cells. Some of this evidence derives from the osteopetrosis mouse model *op/op* that presents a spontaneous mutation leading to the inability to produce the macrophage colony-stimulating factor (M-CSF) and consequently to the absence of macrophages [18]. *Op/op* mice display a number of developmental abnormalities, accompanied by a major reduction in the β-cell mass in both foetuses and adults, defects in β-cell proliferation and islet morphological abnormalities [19]. Interestingly, the α-cell mass in *op/op* mice is not affected, suggesting that the role of macrophages is crucial for the establishment of the mass of insulin-secreting cells, but is dispensable for the development of glucagon-secreting cells [19]. The signalling mechanisms by which macrophages lead to β-cell differentiation still need to be elucidated. However, it was shown that macrophages presenting a foetal M2 phenotype drive the embryonic pancreatic epithelium to exit cell cycle and migrate, promoting endocrine differentiation and the appearance of PDX1^+^ pancreatic progenitors [20]. In addition, the treatment of embryonic pancreatic explants with M-CSF was shown to induce a drastic increase in the development of insulin-secreting cells [21].

**Figure 1:**
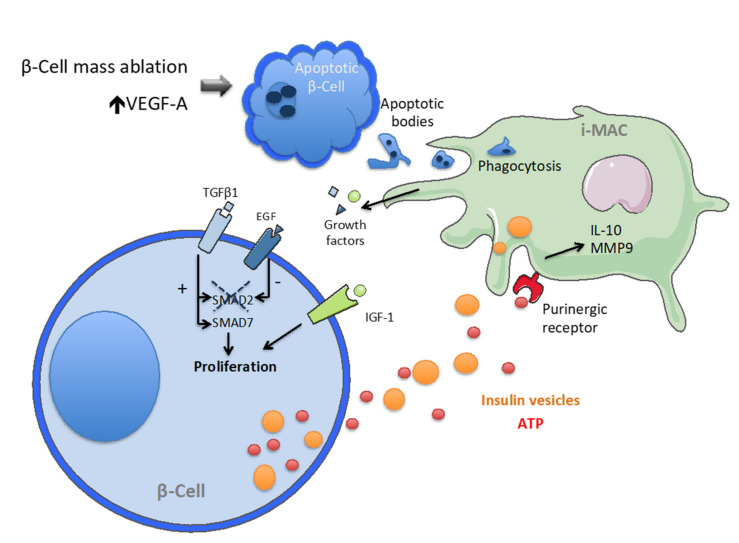
Homeostatic crosstalk between pancreatic β-cells and islet macrophages (i-MAC). Phagocytosis of apoptotic bodies leads to the production of pro-proliferating growth factors (TGFβ1, EGF and IGF-1). Insulin and ATP released by β-cells in response to physiological glucose fluctuation are sensed by macrophages and induce the production of homeostatic signals (IL-10, MMP9).

Macrophage populations in mouse foetal and adult pancreases were analysed, revealing age-related differences in number and phenotype [21]. In mice, F4/80^+^ macrophages are first observed in the pancreas at E14.5. Flow cytometric analysis for surface markers and gene expression profiling in adult pancreases showed that islet resident macrophages have a myeloid origin, namely F4/80^+^/CD11b^+^ with concomitant expression of CD11c, and their phenotype appears to be skewed towards a M1 profile with TNFα and IL-1β expression [22]. This is a peculiar profile, since exocrine pancreas macrophages show an M2-like phenotype. This suggests that adult islet macrophages may hold distinctive functions, specific to the islet microenvironment. In accordance with this hypothesis, different studies reported the role of macrophages in β-cell proliferation and regeneration. In one study, Riley and colleagues analysed the mechanism of β-cell mass regeneration elicited by the connective tissue growth factor (CTGF/CCN2) in a 50% β-cell ablation mouse model [23]. CTGF is a protein associated to the extracellular matrix. Besides inducing intrinsic changes in β-cells, such as the upregulation of cell cycle regulatory genes [23], the authors found that CTGF leads to an increase in islet macrophages. They demonstrated that the expansion of the macrophage population is required for CTGF-induced β-cell proliferation; indeed, the effect of the growth factor was completely abrogated after injections of clodronate liposomes that induce macrophage death [24]. Induction of macrophage populations was also observed following β-cell specific VEGF overexpression. While the vascular endothelial growth factor-A (VEGF-A) released by endothelial cells is necessary during islet embryonic development, it induces major β-cell loss in adult islets. Macrophages appear to be necessary to reconstitute β-cell proliferation in this model [25]. Another report showed that the pro-regenerative action of macrophages is target-specific. After exposure to apoptotic endocrine cells, macrophages undergo a switch in the activation state, resulting in the expression of TNFα, IL-6, and IL-10, and promote the regeneration of this specific cell type from the embryonic pancreatic epithelium, rather than promoting acinar cell development [26]. Recently, it was described that islet macrophages are the main source of insulin-like growth factor 1 (IGF-1), which is secreted following β-cell death, inducing β-cell proliferation and promoting their viability [27] (Figure 1). Other growth factors released from macrophages include the transforming growth factor β (TGFβ1) and the epidermal growth factor (EGF). Following β-cell ablation, TGFβ1 is released by macrophages that switch to a reparative state. TGFβ signalling modulates the R-SMAD protein family, which in turn controls nuclear gene transcription. Paradoxically, TGFβ1 induces SMAD2 phosphorylation and translocation into the nucleus that negatively impacts the cell cycle. However, TGFβ1 simultaneously activates, as negative feedback, the inhibitor SMAD7. Interestingly, the concomitant release of EGF inhibits SMAD2, thus allowing the induction of SMAD7 without impacting the cell cycle (Figure 1). Indeed, SMAD7 seems to have pro-proliferative functions independent of its role as SMAD inhibitor [28] (Figure 1). Altogether, these findings suggest that macrophages are highly sensitive to signals, indicating the viability of pancreatic islet cells, and respond to these cues modulating their activation state and releasing proliferative factors. This has major implications in the pathogenesis of both T1D and T2D and will be discussed in the following paragraphs.

Although the role of macrophages in maintaining β-cell mass is now well established, the mechanisms underlying the crosstalk between these cell types remain mostly uncharacterised. It was shown that purinergic receptors located on mouse islet macrophages allow a precise sensing of the level of ATP in the islet microenvironment [29] (Figure 1). In pancreatic islets, ATP is uniquely released by β-cells together with insulin following high glucose stimulation [30,31]. The activation of purinergic receptors by ATP induces calcium responses in macrophages, changes in gene expression, and increased macrophage motility. In addition, macrophages can capture insulin-containing vesicles secreted by β-cells [32]. Interestingly, these observations were recently confirmed using human living pancreatic tissue slices [33]. Functional studies of human islet macrophages are generally challenging because in vitro culture of islets leads to leukocyte depletion and isolated macrophages rapidly change their phenotypes. The experimental setting of living pancreatic tissue slices overcome these limitations allowing the maintenance of physiological structures and conditions. In their study, Weitz and colleagues reported that human islet macrophages are localised in close proximity to blood vessels and secrete homeostatic factors, such as IL-10 and the metalloproteinase MMP9 [33]. Altogether, the described evidence supports the hypothesis that islet macrophages constantly analyse the composition of their milieu in order to sense the secretory activity of β-cells and to control tissue homeostasis.

## 4. Role of Macrophages in Type 1 Diabetes

Type 1 diabetes (T1D) arises from local pancreatic islet inflammation that contributes to the progressive loss of insulin-secreting β-cells. Although the adaptive immune system and, in particular, T-cells are the major contributors to this process, it is now clear that mediators of innate immunity hold a crucial role during the induction of insulitis [1,34].

The possible role of macrophages was already suggested long ago by means of observations in rodent models of autoimmune T1D. Silica administration, resulting in highly specific macrophage depletion, has been shown to be effective in preventing diabetes development in BB rats [35] and nonobese diabetic (NOD) mice [36]. In NOD mice, this was further confirmed by inhibition of macrophages with a selective monoclonal antibody treatment that prevents islet infiltration and diabetes onset [37]. In the same model, it was shown that pre-diabetic islets produce macrophage-attractant chemokines such as CXCL10, CCL2, CCL20, and IL-15 [38,39]. The increase in IL-1, CCL2, and CCL7 was also shown in serum from T1D patients even before clinical manifestation of the disease [40]. Following chemokine production, macrophages derived from pro-inflammatory circulating monocytes may infiltrate the islets in the initial phases of insulitis. Because the majority of the initial events occur within the islet microenvironment, confirmation of these mechanisms in humans is challenging. However, the characterisation of islet immune cells infiltrates in post-mortem pancreatic sections of T1D patients showed that macrophages (CD68^+^) are present in both early and advanced insulitis [41]. Interestingly, a recent study using multiplexed in situ imaging mass cytometry confirmed the increase in macrophages in T1D patient-derived pancreatic sections and localised them in close proximity to islets still containing β-cells [42].

**Figure 2:**
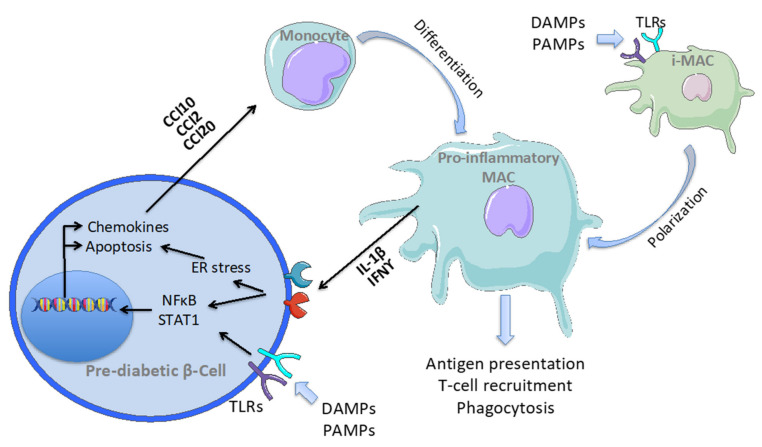
β-cell–macrophage communication during insulitis in T1D. The initiator events occur in still functional pre-diabetic β-cells, when pathways promoting islet inflammation are triggered. The initiation of insulitis in T1D is driven by initiator stimuli, such as damage-associated molecular patterns (DAMPs) and pathogen-associated molecular patterns (PAMPs) or cytokines (IL-1β and IFNγ) binding to specific receptors on the surface of β-cells and inducing endoplasmic reticulum (ER) stress and NFκB- and STAT1-dependent pathways. This leads to the activation of apoptotic pathways and the expression of chemokines. Chemokines attract pro-inflammatory monocytes from the circulation that differentiate into islet macrophages which, in turn, release cytokines targeting β-cells. Apoptotic bodies and TLR ligands may also induce pro-inflammatory polarisation of islet resident macrophages. Pro-inflammatory macrophages mediate T-cell recruitment via antigen presentation and finally phagocytose damaged β-cells.

It has been shown that β-cells themselves can secrete chemokines following exposure to the pro-inflammatory molecules IL-1β and IFNγ or viral infection, and activate NFκB and STAT1 transcription factors [1] (Figure 2). Once inside the islets, macrophages release pro-inflammatory cytokines that have been reported to activate NFκB-dependent pathways in β-cells and induce apoptosis [43,44]. Moreover, macrophages take up insulin-containing vesicles derived from β-cells and may participate in cytotoxic T-cell recruitment via antigen-presenting mechanisms [45,46]. The phagocytic activity of macrophages appears to also be crucial in the final phases of insulitis. In this case, circulating monocyte-derived macrophages are essential for the irreversible decrease in β-cell mass that leads to diabetes development. This was demonstrated using the NOD.scid mouse model, in which the development of T and B cells is impaired and autoimmune diabetes can be triggered by inoculation of activated T-cells. In vivo depletion of macrophages with clodronate liposomes does not block islet infiltration in NOD.scid mice inoculated with activated T-cells, but markedly reduces the incidence of diabetes [47].

Altogether, the evidence described above supports a model of T1D pathogenesis in which macrophages derived from circulating monocytes are the first immune cells to sense the pro-inflammatory microenvironment in pancreatic islets and exacerbate the condition releasing cytokines, leading to T-cell recruitment; finally, macrophages may play a central role in β-cell mass destruction (Figure 2). However, additional evidence in humans must be collected to support these observations. Moreover, the role of islet resident macrophages in T1D insulitis is still unclear and under investigated. As previously described in this review, islet macrophages act as sentinels in sensing β-cell secretory function and capturing cellular content released during cell death, rapidly switching their activation state. For these reasons, islet resident macrophages may play a central role in the initial instauration of a pro-inflammatory microenvironment within the islets, that in turn drives immune infiltration and insulitis. One report showed that depletion of resident macrophages in 3-week-old NOD mice using a monoclonal antibody against the CSF-1 receptor leads to a reduction in infiltrating T-cells and significantly decreases diabetes development [48]. In a very recent report, single-cell analysis was performed in order to characterise the cell types present during the progression of insulitis in NOD mice [49]. Bulk RNA sequencing was performed in the macrophage population sorted from islets of 2-, 4-, 8-, and 12-week-old NOD mice to gather deep transcriptional data. This analysis showed an upregulation of genes downstream of IFN-γ signalling and of IL-2/STAT5 and IL-6/STAT3 pathways together with the progression of the disease [49]. Most of the activated genes were involved in the antigen presentation and production of pro-inflammatory cytokines. While bulk RNA sequencing pointed to a major pro-inflammatory shift in macrophage activation state, single-cell sequencing revealed the presence of five main subsets of macrophage populations expressing specific markers. Specific transcriptomic signatures were found for two subpopulations with stable features throughout disease progression and self-renewal capacity (Mac-1;Apoe and Mac-5;Stmn1), two sets of macrophages with increased pro-inflammatory activity (Mac-2;Atf3 and Mac-3;CXCL9), and one presenting increased apoptotic cell clearance, efferocytosis, and anti-inflammatory properties (Mac-4;Prdx1) [49]. This very advanced work suggested that despite an overall pro-inflammatory progression of the macrophage phenotype during insulitis, these cells may exert several different functions.

The priming factors leading to a pro-inflammatory islet microenvironment remain to be elucidated. Viral infection and cytokines have been shown to initiate the inflammatory responses by inducing the secretion of chemokines from β-cells and triggering β-cell apoptosis [50,51,52]. These inflammation initiators trigger the endoplasmic reticulum stress inside the β-cells, which is a crucial effector pathway mediating apoptosis [53,54,55]. Double-stranded RNA is produced during the viral cycle and is part of pathogen-associated molecular patterns (PAMPs); on the other hand, cellular content released during cell death constitutes damage-associated molecular patterns (DAMPs). These signals are perceived by toll-like receptors (TLR) (Figure 2). β-cells possess TLR2, TLR3, TLR4, and TLR9 receptors [56,57,58] that, once activated, trigger NFκB- and STAT1-dependent pathways [59,60]. Moreover, it was shown that islet-resident macrophages express TLR2, TLR4, and TLR6, and TLR ligands trigger the production of IL-1β, IL-6, and TNFα [61]. Additional studies are required to clarify whether these signals induce a switch in islet macrophage activation and whether this mechanism participates in the initiation of insulitis in T1D.

## 5. Macrophage Signalling in Obesity-Dependent Inflammation and Type 2 Diabetes

Type 2 diabetes (T2D) may develop as a consequence of metabolic risk factors widely diffused in the modern society, such as obesity and overnutrition [62]. These conditions are stressful for the organism, and the activation of low-grade inflammation is triggered as an attempt to adapt to the increased energy load [63]. However, chronic activation of innate immune responses can lead to deleterious effects. Monocytes and macrophages are the major cell types involved in obesity-dependent inflammation. Obesity-related inflammation is traditionally associated with the adipose tissue, where free fatty acids and gut-derived lipopolysaccharides induce a macrophage pro-inflammatory profile [64,65]. In the adipose tissue, adipocytes exert a crucial role in maintaining the organism’s energy balance: they sense insulin released by β-cells after nutrient intake and respond to the hormone by storing nutrients such as triglycerides and glycogen. As a consequence of food intake, adipocytes secrete leptin, an hormone that reduces food intake in a negative feedback loop, and increases lipolysis and thermogenesis controlling the energy balance [65]. Adipose tissue macrophages play an important role in homeostasis and regulate adipocyte insulin sensitivity. However, in the case of overnutrition, macrophages are skewed to a pro-inflammatory profile. Adipose tissue inflammation causes the disruption of the physiological response to insulin, leading to insulin resistance and, consequently, to systemic hyperglycaemia [66]. Interestingly, a recent study demonstrated that adipose tissue macrophages from obese mice secrete exosomes that are able to target muscle and liver cells [67]. In their study, Ying and colleagues showed that exosomes derived from adipose tissue macrophages of obese mice and injected into lean mice induce glucose intolerance, insulin resistance and increased glucose-stimulated insulin secretion [67]. This endocrine function appears to be mediated by the transfer of microRNAs that modulate insulin sensitivity.

**Figure 3:**
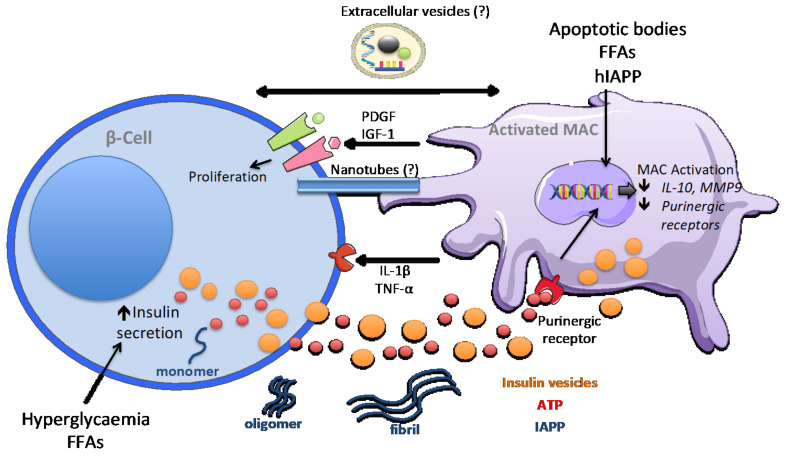
β-cell–macrophage crosstalk in obesity and T2D. Under obesity conditions, β-cells try to compensate over nutrition and hyperglycaemia by releasing more insulin. Macrophages sense the changes in β-cell function via the uptake of insulin vesicles and the hyperactivation of purinergic receptors. This, together with the action of free fatty acids (FFAs), β-cell apoptotic bodies, and hIAPP oligomers and fibrils produced in obese islets activates intracellular processes leading to gene expression modulation. Transcriptional reprogramming triggers the switch in macrophages activation, causing a decrease in IL-10 and MMP9 homeostatic factors and purinergic receptors. Obese macrophages release both growth factors inducing β-cell proliferation and pro-inflammatory cytokines, with a deleterious impact on β-cells. Other proposed routes of communication are tunnelling nanotubes for a direct exchange of cytosolic material and extracellular vesicles.

Increasing evidence demonstrates the occurrence of inflammation and macrophage expansion also in pancreatic islets of T2D patients [68,69] and of rodent models of obesity and T2D [27,70,71]. These observations are supported by clinical evidence that inhibition of IL-1β in T2D patients improves glycemia and C-peptide secretion [72]. Although the identification of the inflammatory signals that trigger T2D pathogenesis remains challenging, it was observed that exposure of human islets to high glucose and free fatty acids (FFA) in vitro induces the production of IL-1β, TNF-α, IL-6, IL-8, and the chemokine CXCL1 [69,73,74]. The FFA-induced islet inflammation profile was inhibited by IL-1 receptor type I (IL-1RI) blockade [73]. Moreover, one of the most characterised triggers of IL-1β production within pancreatic islets is the islet amyloid polypeptide (IAPP), a 37 amino acid hormone secreted by β-cells together with insulin. IAPP controls post prandial blood glucose levels by slowing gastric emptying and digestion with a consequent decrease in food intake. IAPP molecules can aggregate and form plaques and fibrils, which are found in T2D patients and are associated with increased β-cell death [75]. Interestingly, IAPP oligomerisation occurs in human, but not in mice or rats [76,77]. It was shown that in transgenic mice expressing human IAPP (hIAPP), the phenotype of islet macrophages is skewed to a pro-inflammatory profile, and these cells were the major contributors to pro-inflammatory cytokine production [78] (Figure 3). In addition, clodronate-mediated depletion of macrophages improved glucose-tolerance and inhibited inflammation in hIAPP transgenic mice.

Overnutrition induces an initial increase in insulin secretory activity of β-cells in attempt to balance the systemic hyperglycaemia. As previously described, islet macrophages are very sensitive to fluctuation in ATP and insulin levels [29]. In mice fed with a high-fat diet and in T2D patient-derived islets, the expression of purinergic receptor genes *Il-10* and *MMP9* was down-regulated [33]. This suggests that high insulin demand and production present in obesity and pre-diabetes may lead to purinergic receptor over-activation and finally to their desensitisation and to the downregulation of homeostatic signals.

The expansion of islet macrophages in obesity conditions is accompanied by a transcriptional reprogramming. Nackiewicz and colleagues reported an increase in islet macrophages in 8-week-old obese and diabetic *db/db* mice. Gene transcription analysis showed the induction of *Igf1* and reduction in *IL6* and *Tnf* gene expression, supporting the hypothesis that obesity triggers a switch to a macrophage reparative state, probably in response to increased β-cell death [27]. Indeed, the same gene expression profile was observed after administration of streptozotocin, an antineoplastic agent with specific toxicity for pancreatic β-cells, alone or in combination with a high-fat diet (HFD) [27]. Another recent report confirmed the expansion of islet macrophages in HFD-induced obesity and in *db/db* mice [71]. In this report, transcriptomic analysis unveiled a peculiar macrophage activation profile, in which only some pro-inflammatory genes were upregulated, whereas others were even downregulated. In addition, the expression of a subset of M2 macrophage-specific genes was decreased. These observations demonstrate that islet macrophages are highly specialised and present specific phenotypes in response to their microenvironment, rather than a classic pro-inflammatory activation profile. Ying and colleagues further analysed the effect of islet macrophage expansion in obesity [71]. Co-culture with macrophages derived from obese mice impaired glucose-stimulated insulin secretion from clonal Min6 β-cells [71]. This may be mediated by the uptake of insulin-containing secretory vesicles released from β-cells. It has been proposed that direct contact between macrophages and β-cells is required for this mechanism, maybe via tunnelling nanotubes transporting cytoplasmic material [79] (Figure 3). In addition, macrophages isolated from obese mice show increased β-cell proliferation in a mechanism dependent on PDGF-receptor activation [71].

Although we are now beginning to unveil the role of islet inflammation in obesity and T2D, the initial stimuli triggering inflammation and islet macrophage expansion remain to be defined. Circulating saturated fatty acids and high glucose levels are known to induce pancreatic β-cell dysfunction and apoptosis. This may lead to the release of ATP, chemokines, and apoptotic cell components, which activate TLR signalling in macrophages, as previously described in this review. Whether additional communication routes contribute to the crosstalk between macrophages and β-cells in the islet microenvironment will need to be further investigated.

## 6. Discussion

Altogether, the studies discussed in the present review demonstrate that the contribution of macrophages in pancreatic islet development, homeostasis, and pathogenesis is increasingly acknowledged in the last years. In particular, the intense crosstalk between macrophages and β-cells emerges as being at the core of islet responses to systemic metabolic needs and as an important mechanism initiating islet dysfunction in diabetes pathogenesis. However, different questions still need to be addressed to shed more light on the specificity and complexity of macrophage phenotypes and on their roles in diabetes pathogenesis: (i) what is the specific contribution of self-renewed islet resident macrophages? (ii) What is the level of heterogeneity of the macrophage profile within the islet? (iii) Are there unidentified communication mechanisms that participate in the signalling network between macrophages and β-cells?

*Islet resident macrophages versus circulating monocyte-derived macrophages*: of particular importance is the need to discriminate between the contribution to islet inflammation of islet-resident macrophages and of circulating monocyte-derived macrophages. Analysis of surface markers evidenced specific profiles: similarities and differences are summarised in Table 1. The presence of specific markers allows the isolation of the two subpopulations, and therefore the identification of specific functional and molecular changes. The expression of surface markers can be similar to that of antigen-presenting dendritic cells localised in the islets. For this reason, for cell sorting or staining procedures, it is necessary to use a combination of antibodies. This is crucial because a better understanding of the molecular switch occurring in islet resident macrophages during early pre-diabetic stages of T1D and T2D pathogenesis may allow us to identify and eventually target the initiator events preceding islet inflammation.

*Macrophage polarisation:* another crucial factor to be considered when studying the role of macrophages in diabetes pathogenesis is the switch in the activation state. A pro-inflammatory polarisation appears to be unequivocally linked to the development of T1D. Indeed, the modulation of the macrophage activation state towards an anti-inflammatory M2 profile was shown to have anti-diabetogenic properties. The adoptive transfer of in vitro polarised M2 macrophages protects NOD mice from diabetes onset promoting β-cells survival [84]. Although the environmental cues directing macrophage polarisation are still unclear, the production of reactive oxygen species within the islets and the subsequent activation of TLR signalling is certainly a driving factor. NOD mice unable to produce NADPH oxidase (NOX)-derived superoxide (Ncf1(m1J)) are protected against T1D development, and present an impaired M1 pro-inflammatory activation of islet macrophages with a concomitant increase in M2 polarisation, as indicated by chemokine levels in the sera and the surface markers detected upon islet staining [85]. However, a higher heterogeneity is expected to be present. Indeed, advanced single-cell sequencing in islets from NOD mice identified different subsets of islet macrophages including anti- and pro-inflammatory populations at all stages of disease progression [49]. Similar levels of complexity are expected in T2D-associated low-grade chronic inflammation. In fact, high throughput transcriptome analysis of islet macrophages in obesity-related T2D revealed that the inflammation occurring in islets from obese mice is associated with a very specific macrophage metabolic profile, with intermediate characteristics in between pro- and anti-inflammatory activation [71]. These very recent studies highlight the necessity of a deep transcriptional profiling of islet macrophages to fully characterise their phenotype. Moreover, the involvement of epigenetic factors and posttranscriptional gene expression modulators, such as non-coding RNAs, in macrophage metabolic switch associated with diabetes is so far unexplored. These regulatory molecules may add an additional layer of complexity to the islet macrophage profile.

*Cell-to-cell communication in pancreatic islets:* as emerged from observations gathered in both physiological and pathological conditions, macrophages and β-cells exchange a variety of signals, constantly adapting to the systemic metabolic state and consequent insulin demand. Cytokines and chemokines, hormones and growth factors, insulin-containing vesicles and exosomes constitute the molecular mediators of the crosstalk between the different types of cells residing in pancreatic islet. The cytokines released from macrophages have a major impact on β-cell function and viability. TNFα induces NF-κB activation, resulting in impaired β-cell function [86], and IL-1β and IFN-γ reduce the expression of the endoplasmic reticulum (ER) calcium pump, leading to ER stress and to a decrease in glucose-stimulated insulin secretion [87]. In addition, IL-1β activates the c-Jun N-terminal kinase (JNK) pathway causing β-cell dysfunction and apoptosis [88], and induces the nuclear translocation of Foxo-1 that decreases PDX1 expression [89]. The aforementioned evidence demonstrates that macrophages also release growth factors such as IGF1 and PDGF in an attempt to restore β-cell proliferation in damaging and deleterious conditions such as obesity or injury. Macrophages are activated by chemokines released by β-cells, but also continuously sense the β-cell state by capturing insulin granules, detecting ATP secreted together with insulin and hIAPP oligomers, or via phagocytosis of apoptotic β-cells. It has been extensively reported that β-cells release extracellular vesicles, such as exosomes, containing proteins, lipids, and nucleic acids. The exosome cargo is altered by cytokines, hyperlipidaemia or hyperglycaemia [90,91,92]. The horizontal transfer of the exosome cargo to surrounding β-cells can transduce danger signals activating intracellular pathways alleviating β-cell dysfunction or inducing apoptosis in response to the specific deleterious condition. In addition, exosomes are crucial mediators of immune cell signalling and, as such, have been extensively studied in the modulation of immune responses and in therapeutic applications [93]. Recently, it was shown that in NOD mice, T-cells release exosomes able to target β-cells and trigger cell death. This mechanism was found to be mediated by the transfer of functional microRNAs to the target cells [94]. Whether exosomes contribute to the crosstalk between islet macrophages and β-cells has not been explored so far. However, in obese mice, adipose tissue macrophages release exosomes targeting even distant cells in muscles and liver transferring microRNAs that can modulate insulin sensitivity in these tissues [67]. This suggests that an exosome-mediated crosstalk may also participate in intra-islet communication between β-cells and macrophages.

## 7. Conclusions

Macrophages have emerged as key players in pancreatic islet development and homeostasis. In addition, the switch in macrophage activation state plays a major role in diabetes pathogenesis. Despite the difficulties in studying them, such as their limited number in physiological conditions, their heterogeneity, and the involvement of other populations of macrophages in diabetes pathogenesis, many advances have been made in recent years in the characterisation of islet macrophages. New rapidly developing single-cell techniques will permit a more detailed and specific investigation of the molecular events controlling the macrophage metabolic switch initiating islet inflammation and of the two-way communication mechanisms between macrophages and β-cells. This promises to shed new light on the aetiology of both T1D and T2D and will hopefully pave the way to the design of better therapeutic approaches to prevent and treat these metabolic diseases.

## Figures and Tables

**Table 1:** Macrophage-specific surface markers and their expression in self-renewing islet-resident macrophages, circulating monocyte-derived macrophages and dendritic cells in pancreatic islets.

	Islet Resident Macrophages	Monocyte Derived Macrophages	Dendritic Cells	Ref.
MHCII	+++	+++	+++	[71,80]
F4/80	+++	+++	−	[71,81]
CD11c	++	++	++	[61,78,82]
CD11b	++	++	+	[82,83]
Ly6C	−	++	−	[83]

Levels of expression are indicated as absent (−), low (+), medium (++) and high (+++).

## Data Availability

Not applicable.
